# Balancing health and socioeconomic impacts: A uniform framework for evaluating non-pharmaceutical interventions

**DOI:** 10.1371/journal.pone.0324232

**Published:** 2025-06-02

**Authors:** Matjaž Gams, Anže Marinko, Nina Reščič, Aljoša Vodopija, Sophie Vandepitte, Delphine De Smedt, Jana Javornik, Franc Strle, Vito Janko, David Susič, Zoja Anžur, Mitja Luštrek

**Affiliations:** 1 Department of Intelligent Systems, Jožef Stefan Institute, Ljubljana, Slovenia; 2 Jožef Stefan Postgraduate School, Ljubljana, Slovenia; 3 Department of Public Health and Primary Care, Ghent University, Ghent, Belgium; 4 Leeds University Business School, Leeds, United Kingdom; 5 Institute of Contemporary History, Ljubljana, Slovenia; 6 Department of Infectious Diseases, Ljubljana University Medical Centre, Ljubljana, Slovenia; University College London, UNITED KINGDOM OF GREAT BRITAIN AND NORTHERN IRELAND

## Abstract

This paper introduces a uniform evaluation framework for assessing the effectiveness of past non-pharmaceutical interventions (NPIs) in managing infectious diseases, taking into account the cultural and social differences between countries. The framework enables quantifying and finding the optimal balance of both the health and socioeconomic impacts of NPIs. The aim is to assist policymakers in understanding which NPIs lead to the optimal balance by highlighting unnecessary costs - the costs that could be avoided while maintaining the same infection rates. To assess the extent of unnecessary socioeconomic consequences experienced by a country during a past epidemic of infectious diseases, we use the following approach. First, we develop a model that predicts the number of infections from NPIs in a country. Second, we estimate the socioeconomic costs (SEC) of the NPIs universally for countries included in the study. Third, we develop a model that prescribes the NPI plans with the optimal trade-off between the number of infections and the SEC. Fourth, we create a model that specifically adjusts each country’s SEC. Finally, we provide additional analysis to increase comprehension of the effects of NPIs. Demonstrated through an analysis of COVID-19 pandemic responses in 17 countries, the study offers a systematic presentation of the framework and a concrete examination of the integrated effects of NPIs. It provides insights into interventions’ direct and indirect consequences, offering guidance for future epidemic responses. The framework enables a systematic understanding of the effects of the NPIs applied, acknowledging the national diversity in health measure acceptance and implementation. This allows for fair analysis across countries, identifying and displaying the economic, social, and health-related costs of suboptimal NPI strategies, i.e., unnecessary costs. The framework is applicable for any infectious disease, NPIs, or country, assuming the medical interventions are similar, e.g., timing and amount of vaccination.

## Introduction

When dealing with dangerous infectious diseases like flu or SARS-CoV-2 virus, national governments apply various non-pharmaceutical interventions (NPIs) such as obligatory face coverings and lockdowns. These NPIs reduce the number of infections [1,2], but can incur a significant economic cost to the country/region. In addition, they cause a “social cost", e.g., curtailment of freedoms, reduction of social engagement, and anxiety. These effects can also cause negative political consciences, e.g. political resistance or unrest, or decrease the popularity of reigning political parties.

In general, when selecting appropriate intervention plans, policymakers have to carefully balance the predicted NPIs’ benefits against their socioeconomic cost (SEC). This task is challenging, as both the expected benefits and SEC are not easy to estimate. As a consequence, the number of infections sometimes can rise more quickly than anticipated, and on other occasions, there can be a backlash from the public against the NPIs due to their SEC. As empirically demonstrated, these interrelations often result in frequent changes in intervention plans, making it difficult for businesses and citizens to follow and anticipate actions they need to take to comply with the NPIs. In any infectious disease, the same schema appears, it was just highlighted in the COVID-19 pandemic to the point it attracted researchers from various fields, including artificial intelligence (AI), into multidisciplinary teams. This cooperation enables the introduction of new concepts such as searching for best NPIs in advance [3,4] or a uniform framework for past evaluations, presented in this paper.

A tool for finding good intervention plans that takes into account the SEC requires some SEC data and knowledge of the SEC. The overall economic effect can be estimated from economic data collected by most countries. However, disentangling the impacts of the individual NPIs and other factors that influence the economy might be difficult. The social element can be estimated by surveying the public, but such data does not systematically exist on a large scale. This represents one of the substantial barriers to using computational tools for intervention planning.

This framework assumes that the policy-makers attempted to optimize the trade-off between the benefits of NPIs and their SEC so that the intervention plans they prescribed and tweaked over time reveal the estimated SEC for their country. For example, suppose a country never implemented obligatory facial coverings. In that case, one can reason that the policy-makers believed that this NPI carries a significant SEC, probably social since the economic cost for this particular NPI can be estimated quite reliably. In our analysis, we optimise the trade-off between two conflicting objectives: the number of infections and SEC. We do not prioritise SEC over the number of infections or vice versa. Instead, we fix the number of infections to what was recorded in reality and aim to identify NPIs that could achieve the same infection outcomes while minimising SEC. This approach allows us to analyse how NPIs can be optimised to be more effective economically and socially (we compute unnecessary SEC) without compromising public health outcomes.

A short description and motivation for the framework are presented here: the framework starts by defining a fixed SEC for each NPI, called “uniform", taken from our previous study [4]. By computing the best NPI schedules and the used ones it is possible to compare the cost-effectiveness of the intervention plans produced by our tool with the applied ones in real life. Since the computed best NPIs balance the benefits and SECs of NPIs and the infection rates (first when using the uniform SECs) and humans use their knowledge without optimizing tools, differences are to be expected in the form of “unnecessary costs". The uniform SECs are then adjusted to the new country-specific SECs by computing modifications from those implied by the real-life intervention plans for different countries. Best NPI plans are computed and compared again. Then, intervention plans are analyzed under different SECs from various perspectives to improve the understanding of the SECs’ impact. This framework was conducted on 17 developed countries for which high-quality data is available, from 1st of March 2020 to 28th of April 2021.

As this work is an extension of our previous research, we outline the added value of this manuscript. The infection prediction model, SEC uniform costs, and the NPI prescriptor were part of our previous work [4] and are not novelties in this paper. However, two important contributions distinguish this work from the prior studies are:

- A framework to adjust the uniform costs to be country-specific based on the country’s history of implemented NPIs, which allows for a more nuanced, culturally-aware analysis of the NPIs implemented during the past pandemic.
- A framework for the evaluation of the unnecessary SEC before and after country-specific adjustments, which provides valuable insights into both the direct and indirect consequences of interventions and offers key guidance for future epidemic responses.

These concepts are applied in the COVID-19 case study, where we analyze 17 countries to compare real-world NPI strategies and their associated costs.

### Related work

Before COVID-19, research on interventions strategies for controlling pandemic influenza was already established. Notably, Halloran *et al*. [5] used stochastic simulations to evaluate strategies like antiviral treatment, quarantine, school closures, and social distancing. Their “targeted layered containment” approach showed that timely implementation of interventions could significantly reduce illness rates before vaccines became available, providing key insights for later COVID-19 NPI studies. Similarly, Smith *et al*. [6] used a computable general equilibrium model to assess the economic impact of pandemic influenza and mitigation strategies, finding that interventions like school closures and antivirals could lead to substantial gross domestic product (GDP) losses.

Following the start of the COVID-19 global pandemic in early 2020, research quickly focused on investigating NPIs to mitigate the crisis. For example, Chinazzi *et al*. [7] studied how travel restrictions influenced the spread of the pandemic on both national and international levels using a global meta-population disease transmission model and showed that travel restrictions had a greater effect globally than locally. Prem *et al*. [8] proposed a framework for implementing physical distancing measures, while Lai *et al*. [9] evaluated various NPIs by modeling outbreaks and intervention scenarios using human movement data. Similarly, Pan *et al*. [10] examined the combined effect of public health interventions in controlling the outbreak in Wuhan, China.

As the pandemic spread, researchers also examined NPIs across different countries. For example, Flaxman *et al*. [1] used a Bayesian hierarchical model to estimate infections and deaths in 11 European countries, showing that NPIs led to a decrease in disease transmission. Hsiang *et al*. [11] employed econometric methods to analyze NPI effects across six countries, highlighting how NPI effectiveness varied across cultures and how each intervention carried a certain social and economic cost. In addition, Segarra-Blasco *et al*. [12] used data from 59 countries to assess how nations tailored their NPI strategies to mitigate socioeconomic impacts.

Research also addressed the social impacts of NPIs. For example, Schneiders *et al*. [13] found that NPIs had mostly negative effects on mental health and well-being, while Claes *et al*. [14] showed that subjective well-being decreased, particularly in younger populations. Khan *et al*. [15] highlighted how the pandemic disrupted healthcare services for cardiovascular patients, emphasizing the need for better coordination between policymakers and healthcare systems.

The problem of selecting appropriate intervention plans led to a sizable body of research on the effects of individual NPIs [1,2], and semi-automatic policy construction and evaluation using computational tools appeared. Several studies also used computational tools to assist in policy design. For example, Yousefpour *et al*. [16] developed a SEIRD-based multi-objective optimization framework to recommend NPIs. Chen *et al*. [17] explored the trade-off between expected mortality rates and returning to normal activities using linear programming, while Yaesoubi *et al*. [18] created a decision tool to determine when to trigger or stop physical distancing measures.

Finally, in our previous work [3,4], we developed a tool for finding optimal NPI plans using multi-objective optimization. This tool included an infection predictor model and a genetic algorithm-based prescriptor that optimized the trade-off between reducing infections and SEC based on the NPIs implemented. The prescriptor demonstrated significantly better results compared to real-life interventions implemented by policymakers. The mentioned papers are summarized in Table 1.

**Table 1:** Summary of related works.

Reference	Key focus	Year
Janko *et al*. [4]	AI-based optimization of NPI strategies to reduce infections.	2023
Claes *et al*. [14]	Social impacts of NPIs, subjective well-being decreased, particularly in younger populations.	2023
Khan *et al*. [15]	Pandemic’s impact on healthcare services for cardiovascular patients.	2023
Schneiders *et al*. [13]	Cross-country qualitative study of NPI effects on mental health and well-being.	2022
Moore *et al*. [2]	Analysis of vaccination and NPIs for pandemic control.	2021
Segarra-Blasco *et al*. [12]	Analysis of government economic responses to NPIs across 59 countries.	2021
Chen *et al*. [17]	Trade-off between expected mortality rates and returning to normal activities using linear programming.	2021
Yaesoubi *et al*. [18]	Decision tool for determining when to trigger or stop physical distancing measures.	2021
Flaxman *et al*. [1]	Impact of NPIs on transmission rates using Bayesian hierarchical models.	2020
Hsiang *et al*. [11]	Effects of NPIs on COVID-19 in six countries using econometric analysis.	2020
Lai *et al*. [9]	Evaluation of various NPIs by modeling outbreaks using human movement data.	2020
Pan *et al*. [10]	Evaluation public health NPIs in controlling outbreaks in Wuhan, China.	2020
Yousefpour *et al*. [16]	Multi-objective optimization framework for recommending NPIs using SEIRD models.	2020
Prem *et al*. [8]	Framework for implementing physical distancing measures to reduce infection rates.	2020
Chinazzi *et al*. [7]	Modeling global and local effects of travel restrictions on COVID-19 spread.	2020
Smith *et al*. [6]	Economic impact of pandemic influenza and mitigation strategies using a computable general equilibrium model	2011
Halloranet al. [5]	Individual-based stochastic simulations of pandemic influenza interventions	2008

For a systematic review of empirical studies comparing the effectiveness of NPIs against COVID-19, see Mandez-Brito *et al*. [19]. For a comprehensive review of related work on models that combine epidemiological and macroeconomic projections to help policymakers balance both health and macroeconomic objectives, see Bonnet *et al*. [20].

### Dictionary and structure

Due to the novel use of several concepts, a short dictionary is presented:

- Country-specific stringencies or subjective stringencies - Adjustments in SEC calculations based on each country’s unique context and NPI response.
- Multi-objective optimization - A process to find solutions that balance multiple conflicting objectives, such as health outcomes vs. economic costs.
- Non-pharmaceutical interventions (NPIs) - Strategies implemented to control the spread of infectious diseases without using medication or vaccines, e.g. closure of schools.
- Pareto front - A set of non-dominated solutions, representing the best possible trade-offs in multi-objective optimization.
- SEIRD model - A mathematical model predicting infections based on susceptible, exposed, infectious, recovered, and dead population compartments.
- Socioeconomic costs (SEC) - The economic and social consequences of implementing NPIs.
- Strictness - The degree of enforcement of an NPI, say 0 to 4. Strictness of zero means that the NPI is not enforced at all.
- Stringency - Corresponds to the associated cost based on the active NPI’s social and economic impact, e.g. the cost of an NPI at strictness equal to one.
- Uniform costs - Standardized estimates of SECs for comparison across different countries.
- Uniform/country-specific - Uniform in this paper denotes the same for all countries while country-specific or “subjective" means adjusted for country specifics.
- Unnecessary cost - The difference between the cost of the applied NPIs and the cost of the optimal NPIs resulting in the same infection rates.

The rest of the paper is structured as follows. Section Methodology details the algorithms used to search for the optimal NPI schedules under uniform costs, including adjustments for country-specific stringencies. In section Case study: COVID-19, we demonstrate the evaluation framework with uniform SECs using COVID-19 data. Section Results presents the results of the framework application on 17 countries, comparing real-life versus optimal unnecessary uniform and country-specific costs, and adding a study with COVID-19 deaths. The discussion and conclusions are provided in the final section.

## Methodology

The objective of this study is to assess the extent of unnecessary socioeconomic consequences experienced by a country during a past epidemic of infectious disease, utilizing an analysis of the NPIs employed during that period. To achieve that, we need 1) a model that predicts the number of infections from NPIs in the country; 2) an estimate of SECs of the NPIs; 3) a prescription model that finds the prescription plans with the optimal trade-off between the number of infections and the SEC; and 4) a model that adjusts the uniform SEC for each country based on their unique cultural orientations.

### Predicting the infections

**Fig 1:**
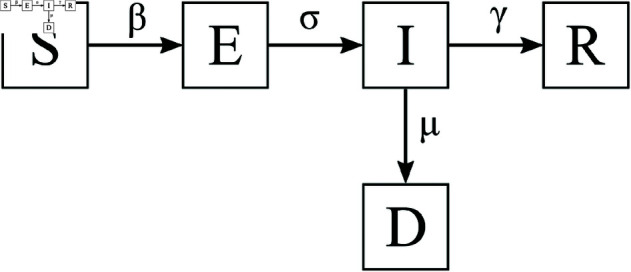
Scheme of the SEIRD model.

The infection prediction model used in this study is a composition of a SEIRD epidemiological model [21] and supervised machine-learning (ML) regression models. SEIRD is a system of coupled differential equations that describe the dynamics between five compartments of a country’s population: susceptible (S), exposed (E), infectious (I), recovered (R), and dead (D). The diagram of the SEIRD model is shown in Fig 1. Parameters β, σ, γ, and μ denote the infection rate, incubation period (1/days), recovery rate (1/days) and mortality rate (1/days), respectively. To train the prediction model, first, the SEIRD parameters β, σ, and μ are fitted to different territory/time intervals by minimizing the difference between predicted and reported infections and deaths, while the recovery rate γ is fixed. This is because γ is generally consistent across countries and contexts, and is usually well-established early in an epidemic (e.g., ~1/5 days in the case of COVID-19 [22,23]). Fixing γ simplifies the learning task and helps avoid identifiability issues when estimating the other, more variable parameters. Second, the fitted values are then used as prediction targets for three separate supervised ML regression models (one for each of the β, σ, and μ parameters), specifically using linear regression, and trained on a variety of predictor features including NPIs, vaccination data and more.

The prediction of infections for a country for a selected period is thus achieved in two steps. In the first step, the SEIRD epidemiological model parameters are estimated based on the active NPIs and other features using ML models. In the second step, the estimated parameters are input to the SEIRD model to model the infection curve. In this manuscript, we analyse the case study of COVID-19 pandemic. The details of the predictor implementation are given in the Case study: COVID-19 section and in [4].

### Socioeconomic costs

SEC describe the social and economic effects of the individual NPIs implemented based on their strictness and stringency. The strictness describes the intensity with which an NPI is active, e.g., a “school closure” with low strictness means a recommended closure, medium strictness means a partial closure and maximum strictness means that all relevant institutions are closed. The NPIs can have a different number of possible intensity levels, but for all of them, a strictness of zero means that the NPI is not active. Stringency, on the other hand, corresponds to the associated cost based on the active NPI’s social and economic impact. The impacts are calculated as economic and social costs as presented in Eq (1) and Eq (2). The social impact involves perceived strain, dread and loss, perceptions of restricted freedoms, and constraining behaviours, whereas the economic impact corresponds to the GDP loss. Social costs are determined by human behaviour analysis and can only be determined by a pairwise comparison of costs of the NPIs and then ranked from the most to least costly, as opposed to GDP loss, which can be measured in monetary units. For practical implementation, the overall SEC are currently calculated as the weighted average between the social and the economic cost Eq (3); however, can be arbitrarily defined in the uniform framework. The definitions in this paper are generally aligned with real life since twice stricter application of an NPI is usually accompanied by twice more costly consequences.

It is important to understand the framework’s flexibility in accepting diverse inputs and relationships between social and economic costs. The fundamental concept of the framework is to determine the costs of optimal strategies/plans and compare them with those caused by decision-makers to enable future improvements based on past inaccuracies.

economic_cost(NPI, day)=strictness(NPI, day)  economic_stringency(NPI) (1)

social_cost(NPI, day)=strictness(NPI, day)  social_stringency(NPI) (2)

SEC(NPI, day)=f(social_cost(NPI, day),economic_cost(NPI, day)) (3)

The definitions refer to one day only. For longer periods, they are calculated as corresponding sums.

The definition states that the costs are dependent on the fixed stringency (costs at low strictness, which is 1 in this paper) and its strictness on a given day. Stringencies are typically established uniformly for a group of countries that share similarities in geography, education levels, industrial development, wealth, and political structures [19]. Thus, we refer to these costs as uniform costs.

### Prescribing interventions

Prescriptor prescribes the NPI plans. An NPI plan is a set of NPIs and their strictnesses to be used each day over a selected period in a given country. The NPI plan to be implemented in real life should limit infections the most while having as low an SEC as possible. The two objectives conflict (generally, the NPIs that limit the infections the most also have the highest SEC), thus the problem is mathematically a multi-objective optimization problem and the goal of the prescriptor is to find the plans representing the Pareto front, i.e., the optimal trade-offs between the two objectives. The prescriptor used in this study was the evolutionary algorithm Nondominated Sorting Genetic Algorithm II (NSGA-II) [24]. The prescriptor finds solutions by first creating a set (a population) of random NPI plans and then evolving (modifying) that population over time to identify better solutions. Each solution is evaluated by inserting the NPI plan to the predictor model to estimate the number of expected infections and by calculating the plan’s aggregated SEC. A solution is better than the other if it is better according to one objective and at least as good in the other objective. Over time, the population converges into the Pareto front which consists of optimal solutions that cannot be further improved. While the convergence of the population does not guarantee absolute optimality, state-of-the-art evolutionary algorithms, like NSGA-II, are highly proficient in practice. These algorithms excel in identifying solutions that are either optimal or close to optimality. In our case, the algorithm effectively balances the conflicting objectives of limiting infections and minimising SEC, making it reliable for prescribing the best NPI plans and guiding real-world policy decision-makers. Because the algorithm is stohastic, we have ran five trials for each experiment, and then averaged the results. The full details of the implementation of the prescriptor algorithm used in this study are given in [4]. The Pareto front calculation is described in Algorithm 1. Based on the defined maximal strictnesses of the *n* NPIs, $S=\{s_{k}{\}}_{k=1}^{k=n}$, their uniform stringencies, $C=\{c_{k}{\}}_{k=1}^{k=n}$, and the infections prediction model, *P*, the algorithm finds NPI plans with the optimal trade-offs between the number of infections and the SEC. As the solutions are found using an evolutionary algorithm, population size *m* and number of iterations $i_{\text{max}}$ must also be defined. Additionally, a granularity parameter *g* must also be selected which determines for how long the NPIs must be fixed before they can change. This stems from real life where changing NPIs each day is impractical. The number of time steps in an NPI plan, *t*, is a multiple of granularity. In our case, the granularity was set to *g* = 14 days. Each NPI plan can be formulated as a $M_{n\times t}$ matrix, where the *M*_*ij*_-th value corresponds to the strictness of the i-th NPI in the j-th time slot.

**Algorithm 1. Calculate optimal NPI plans for a selected country and a period**.



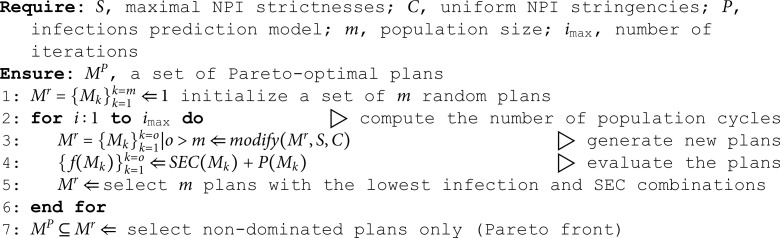



**Fig 2:**
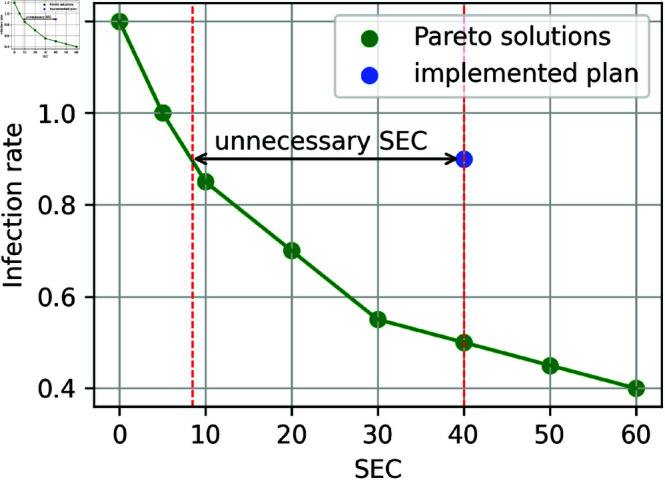
Visualization of the unnecessary SEC. The green points represent the NPI plans with the optimal trade-off between the infection rate and the SEC, whereas the blue point represents the country’s actual implemented plan.

The unnecessary SEC of the NPI plan implemented by the real-world policy decision makers is a difference in SEC between the implemented NPI plan and the plan on the Pareto front with the same number of infections (see Fig 2). It denotes how much lower the SEC could have been in the country during a selected period, assuming the same number of total infections, if the country’s decision-makers had chosen NPI plans found by the genetic algorithm instead of those proposed by humans. The unnecessary SEC is defined as

$$\text{unnecessary\_SEC(period)}=\sum_{\text{day}\in \text{period}}\sum_{\text{NPI}\in \text{NPIs}}(\text{SEC(NPI, day)-minimal\_SEC(day)).}$$ (4)

If the unnecessary SEC is very small, that means that the country implemented an NPI plan that is very close to optimum. It is important to note that by estimating SEC in the described way it is considered that the stringencies of the NPIs are uniform and do not consider the countries’ specific cultural orientations, which may have affected their decision-making in prescribing the NPIs. As an example, there are countries where closing churches is not acceptable under any circumstances. To accommodate for these differences, our studies are performed first on the uniform and later on the country-specific criteria. These two ways of calculating and comparing unnecessary SECs are considered novel and more fair than the previous approaches.

### Country-specific stringencies

The subjective stringency takes into account the country’s past implemented NPIs and adjusts the stringencies to the country’s preferences. The idea behind this approach is based on the assumption that the policymakers attempted to optimize the trade-off between the number of infections and the SEC while simultaneously complying with the country’s unique cultural orientations. For example, when choosing between the closure of companies or schools, the decision makers usually also take into consideration the public unrest caused by each NPI, and since the revolt might be different in different countries, the well-grounded decision might be different in different countries. The decisions taken have their trace in the intervention plans they prescribed and tweaked over time thus revealing the estimated social and economic stringencies for their country. A simple explanation of this phenomenon can be demonstrated in the following way: suppose one person prefers climbing and the other dancing. As a consequence, one will climb more, and the other dance more. Observing only behavior and not being familiar with the preferences, it could be concluded that the first person prefers climbing and the other dancing. This is the supporting hypothesis of our approach since we compute country-specific preferences based on the applied NPIs.

The procedure of estimating the country-specific costs is presented in Algorithm 2. The algorithm finds such stringencies so that the country’s actual implemented plan is as close to the Pareto front as possible. Note that in this case, the Pareto front, calculated using Algorithm 1, also changes concerning the changes in the stringencies. The Algorithm 2 uses the same evolutionary algorithm as Algorithm 1. The solution representation in this case is a $N_{2n\;\times \;t}$ matrix. The column values *i* represent the social (i<=n) and economic (n<i<=2n) stringencies, $C^{s}=\{c_{k}^{s}{\}}_{k=1}^{k=n}$ and $C^{e}=\{c_{k}^{e}{\}}_{k=1}^{k=n}$, whereas *j* represents the time slot. The initial country-specific stringencies are set to the uniform ones. The stringencies are modified over time and evaluated each iteration until termination criteria is met. The stringency from Eq (1) becomes dependent on time and the equation for economic cost becomes

economic_cost(NPI, day)=strictness(NPI, day)  economic_stringency(NPI, day). (5)

Similarly, all the equations change accordingly.

Note that the deviations from the uniform stringencies are limited with $B^{s}=\{b_{k}^{s}{\}}_{k=1}^{k=n}$ and $B^{e}=\{b_{k}^{e}{\}}_{k=1}^{k=n}$ as the country-specific stringencies should not be too different from the uniform ones which are based on literature and expert knowledge and are calculated for a selection of similar countries with reliable data. In addition, the total sum of country-specific stringencies must also not change, meaning none of the countries is preferred, therefore, the algorithm is only allowed to reweigh the stringencies between the NPIs and between the economic/social components.

**Algorithm 2. Find country-specific stringencies so that the country’s past implemented NPI plan is as close to the Pareto front as possible**.



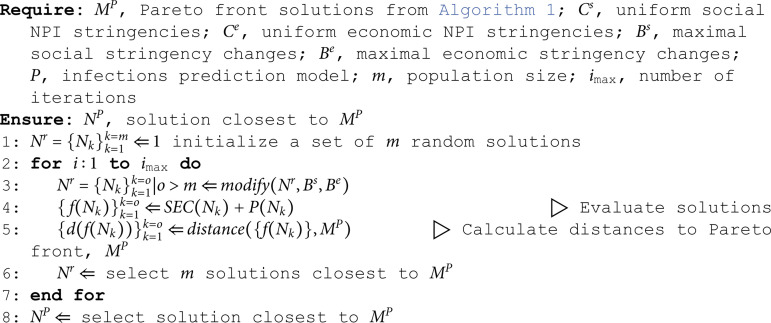



The Algorithm 2 introduces two modifications to calculations of stringencies: first, stringencies are adjusted to correspond to the cultural specifics of each country, and second, stringencies change over time, unlike the static uniform stringencies. From the social impact point of view this makes sense as, over time, people change their preferences, e.g. get tired of some interventions such as “school closing" or “stay at home requirements”, whereas the resistance towards others might decrease, e.g., “facial coverings" applied in the neighbouring country. From the economic impact point of view this also makes sense as industries, shops, and public places, such as bars, restaurants are opened/closed in intervals, which, in most cases, correspond to the country’s infection waves.

## Case study: COVID-19

To demonstrate the performance of the framework, we need to choose particular countries, periods, and an infectious disease. The early stages of the COVID-19 pandemic, i.e., the first three waves, were chosen as the best-studied recent example. For this study, we focused on the core aspects of the problem and avoided introducing additional complexities.

### Data

This paper analyses past implemented NPIs during the COVID-19 pandemic in 17 different countries: Belgium, France, Germany, Israel, Italy, Netherlands, Hungary, Portugal, Czech Republic, Slovenia, South Africa, Spain, Sweden, United Arab Emirates, United Kingdom, and the United States. The scope of the analysis was COVID-19 data from March 1st, 2020, to April 28th, 2021. We focused on 12 NPIs selected from the Oxford-based NPIs, that is OxNPIs database [25]. Some of the policies (economic policies and policies H4, H5, H7, and H8) were excluded, as they do not directly impact the number of infections.

Our dataset included not only NPIs but also various dynamic and static factors to enhance the infection prediction model. These included vaccination data [26], COVID-19 strains [27], daily infections and deaths (sourced from the OxNPIs database [25]), testing rates [28], hospitalizations and intensive care unit (ICU) admissions [29], mask usage data [30], mobility trends [31,32], weather [33], holidays [34], and 93 static country-specific features related to development, culture, and healthcare [4].

To address NPI adherence, we incorporated “duration” features, tracking how long an NPI was active in total since the first time it was activated. This was based on the assumption that adherence might decline over time as populations grew fatigued with restrictions. Additionally, we included behavioral traits - agreeableness, indulgence, openness, and conscientiousness - within the static features to indirectly capture adherence and trust in public authorities, as direct data on trust and compliance were unavailable. Importantly, adherence differs from policy strictness; high adherence can be achieved even under lenient policies if trust in public authorities is strong. In contrast, stricter measures may be necessary in contexts with lower trust, often resulting in higher SEC.

In our analysis, we focused on 17 previously listed WEIRD countries (western, educated, industrialized, rich, and democratic) because these nations exhibit relatively homogeneous economic, cultural, and social structures. This uniformity ensures that the uniform SEC measured across these countries are comparable and minimizes discrepancies that might arise from differences in governance systems or healthcare infrastructures. This methodological approach, commonly used in psychological and behavioral research [35], enabled us to apply the same framework consistently without needing country-specific model adjustments. Additionally, our selection was driven by data availability, as we included only countries with complete datasets across all relevant features to maintain data integrity and avoid uncertainties associated with data imputation.

Finally, while our dataset (and consequently, our models) does not explicitly account for underreporting of infections or deaths, we aim to minimize this limitation by focusing on WEIRD countries with robust surveillance systems and relatively transparent reporting. While some degree of reporting bias may still exist, we expect its effect on our results to be limited due to the overall data completeness and quality in the selected countries.

### Predicting the COVID-19 infections

The β, μ, and σ parameters of the SEIRD epidemiological model were fitted individually for each country by finding values that minimized the least squares error in predicting the reported number of infections and deaths. We acknowledge that this approach assumes Gaussian noise in the discrepancy between observed and predicted values, which may not be ideal for epidemiological count data, especially at low incidence levels. While more appropriate loss functions that align with likelihoods such as Poisson or negative binomial are commonly used in epidemiological modeling to better capture the discrete and overdispersed nature of case data, least squares was selected here due to its simplicity and widespread use in curve fitting within computer science. The γ parameter was fixed to 1/5 days, which is in agreement with other studies [22,23]. The fitted parameter values, based on incidence (i.e., new reported cases and deaths per day), were then used as prediction targets for ML regression models, with a separate model trained for each of the β, μ and σ parameters. Initially, we included all the features described in the previous section as predictor variables. However, during feature selection, we found that the strongest correlations with infection trends were attributed to the NPIs, NPI durations, COVID-19 strains, vaccination data, and historical infection data. To maintain model interpretability and prevent overfitting, we retained only these key features, discarding the others.

Among the various ML models tested, linear regression performed best [4]. This model assigns weights to each NPI based on its learned contribution, allowing us to isolate the effects of individual NPIs despite their implementation in combination. Estimating the impact of individual NPIs is typically challenging since NPIs are applied together, making it difficult to determine their separate effects. Linear regression helped overcome this challenge by providing a clear attribution of NPI impacts. A more complex model than linear regression could model complex interaction between interventions. However, linear regression outperformed them, most likely because it overfits less and this advantage outweighs the disadvantage of not being able to model complex interactions.

The prediction pipeline’s objective is to estimate the number of infections based on a given intervention plan (i.e., the NPIs used each day). A feature vector is constructed by combining the NPI data with the other selected predictor features. Each day, the three model parameters (β, μ, σ) are predicted using the ML models, and these estimates are then fed into the SEIRD model. This allows the model to generate daily infection counts over a defined prediction period. Further details on the prediction methodology are available in [4].

### Socioeconomic costs

Economic stringencies for the countries were derived from the relative share of GDP loss during the implementation period of the NPIs, determined by the sum and reconstructed into specific ones. They are expressed as a relative share of GDP loss during the period when the NPI was implemented. Country-specific GDP values (in US dollars [36]) were used for this calculation. For example, if the “C3 Cancel public events” NPI is active for one month with an associated cost of 0.014, this implies a 1.4% reduction in GDP for that month relative to normal conditions. It is important to note that this percentage reflects the GDP impact for the specific time frame, not an annual loss.

On the other hand, the social component was determined based on the perceived strain, dread and loss, perceptions of restricted freedoms, and constraining behaviors (i.e., on the negative impact of each measure on behavior, attitudes, and one’s well-being) and expressed on a scale of 1–12.

The stringency values used in this study were introduced and explained in more detail in our previous work [4]. They were derived from prior research and expert opinions and were assessed individually [4]. Thus, although NPIs are often combined in practice, our methodology treats them as independent factors. They were calibrated for WEIRD countries. Table 2 presents uniform social and economic SECs for each NPI included in our study, calculated using uniform stringency and maximal strictness, calibrated for WEIRD countries. The last column contains aggregated cost, calculated as the weighted sum of the social and economic costs. It is important to note that the cost of a particular implemented NPI in a given country over the study period varies due to the varying levels of strictness and stringency. As presented in Table 2, “C6 Stay at home requirements” had the highest social cost and “C2 Workplace closing” had the highest economic and aggregated cost. In all tables, the highest values in columns are bold and the lowest in italics. The full description of the calculation OxNPIs SECs is given in [4].

**Table 2:** Uniform social, economic and aggregated cost for the 12 OxNPIs, i.e., uniform stringency multiplied by maximal strictness of each NPI. Economic cost is shown as a relative share of GDP loss in the period the NPI was implemented. The social cost is based on domain knowledge and expressed on a 1–12 scale. The aggregated column is the average of the two costs, when both are normalized to be between 0 and 1.

OxNPI	Social cost	Economic cost	Aggregated cost
C1 School closing	11.0	0.03912	1.10059
C2 Workplace closing	11.0	0.22021	2.93164
C3 Cancel public events	7.0	0.01434	0.59375
C4 Restrictions on gatherings	10.0	0.01434	0.78613
C5 Close public transport	2.0	0.00261	0.15454
C6 Stay at home requirements	12.0	0.05212	1.29688
C7 Restrictions on internal movement	10.0	0.07819	1.43164
C8 International travel controls	2.0	0.06604	0.7959
H1 Public information campaigns	1.0	0.00003	0.06433
H2 Testing policy	1.0	0.00587	0.12341
H3 Contact tracing	1.0	0.00117	0.07593
H6 Facial coverings	10.0	0.00026	0.64355

Table 3 shows uniform SECs of the NPIs included in the analysis for 17 different countries. The SECs were calculated for the same period for all countries using uniform stringency and real-life implemented strictness. As seen in Table 3, when considering “C1 School closing," Belgium had the lowest cost, while the United States had the highest, indicating that this NPI was used most frequently in the United States. The SECs of some NPIs were found to be relatively uniform across countries, such as “H1 Public information campaigns," which were inexpensive and implemented almost continuously across all countries. However, the SECs of other NPIs, such as “C1 School closing," “C6 Stay at home requirements," and “C7 Restrictions on internal movement," varied significantly across countries.

**Table 3:** Uniform aggregated SEC for the 12 OxNPIs in the 17 studied countries over the study period. Italics denote the smallest and bold the largest number in a column. Abbreviations: CR, Czech Republic; UAE, United Arab Emirates; UK, United Kingdom; US, United States.

Country	C1	C2	C3	C4	C5	C6	C7	C8	H1	H2	H3	H6
Belgium	*0.44*	2.09	0.57	0.73	*0.00*	0.61	0.32	0.64	0.06	0.10	0.08	0.24
CR	0.71	1.60	*0.50*	0.60	0.02	0.45	0.78	0.57	0.06	0.11	0.08	0.35
France	0.54	2.03	0.58	0.79	0.02	0.55	0.79	0.58	0.06	0.11	0.07	0.45
Germany	0.72	2.03	0.59	0.74	0.03	0.45	0.97	0.61	0.06	0.11	0.07	0.12
Greece	0.68	1.67	0.58	0.70	0.08	0.67	1.08	0.56	0.06	0.10	0.06	0.35
Hungary	0.69	1.64	0.56	*0.53*	0.04	0.60	0.31	0.72	0.06	*0.09*	0.07	0.14
Israel	0.64	1.96	0.57	0.65	0.07	0.54	0.84	0.69	0.06	0.11	0.07	0.34
Italy	0.90	2.29	0.59	0.64	0.05	0.86	1.33	0.60	0.06	0.11	0.08	0.59
Netherlands	0.54	2.08	0.57	0.70	0.04	0.57	0.39	0.58	0.06	0.10	0.07	0.10
Portugal	0.65	2.13	0.58	0.76	0.07	0.51	*0.30*	0.58	0.06	0.12	0.07	0.19
Slovenia	0.64	1.66	0.58	0.66	0.05	0.43	0.70	0.49	0.06	0.11	0.07	0.34
South Africa	0.56	1.47	0.54	0.55	0.05	0.77	0.60	0.54	0.06	0.12	0.07	0.29
Spain	0.57	1.94	0.58	0.73	0.02	0.70	1.14	0.63	0.06	0.10	0.07	0.42
Sweden	0.47	*1.30*	0.58	0.64	0.07	0.41	0.32	0.60	0.06	0.10	0.07	*0.03*
UAE	0.62	1.43	0.57	0.75	0.04	*0.11*	0.79	0.50	0.06	0.12	0.08	0.35
UK	0.79	2.30	0.57	0.75	0.07	0.58	1.18	*0.36*	0.06	0.10	*0.06*	0.22
US	1.00	2.11	0.58	0.73	0.07	0.78	1.31	0.60	*0.06*	0.10	0.07	0.60

Table 4 presents the uniform social and economic cost of all NPIs implemented in each of the 17 studied countries over the study period. The last column contains aggregated cost (SEC), calculated using uniform stringency. The aggregated column of this table is equal to the sum of the columns in Table 3. According to Table 4, Sweden had the lowest SEC, while Italy and the United States had the highest. The ranking of countries in terms of social and economic costs corresponded to the aggregated costs.

**Table 4:** Uniform social, economic, and aggregated SEC for the 17 studied countries over the studied period. Italics denote the smallest and bold the largest number in a column. Economic cost is shown as a relative share of GDP loss, whereas the social cost is based on domain knowledge. The aggregated cost is the average of the two costs after normalization. Abbreviations: CR, Czech Republic; UAE, United Arab Emirates; UK, United Kingdom; US, United States.

Country	Social cost	Economic cost	Aggregated
Sweden	*34.78*	*0.24*	*4.64*
UAE	43.00	0.26	5.42
Hungary	40.81	0.28	5.45
South Africa	44.69	0.27	5.61
Slovenia	46.56	0.28	5.80
Netherlands	41.75	0.31	5.80
CR	45.84	0.29	5.83
Belgium	43.84	0.30	5.88
Portugal	45.69	0.31	6.04
Germany	48.03	0.34	6.51
Israel	50.28	0.33	6.56
France	51.34	0.32	6.58
Greece	52.22	0.32	6.59
Spain	53.78	0.35	6.95
UK	53.81	0.36	7.05
US	64.88	0.38	8.02
Italy	63.97	0.40	8.10

Sweden’s NPIs were implemented with low strictness, resulting in the lowest economic costs among the studied countries. Additionally, Sweden’s high trust in government and institutions significantly reduced social costs, contributing to its low overall SEC. This unique combination of low strictness and high trust was context-specific and may not be replicable elsewhere. In contrast, Italy faced rapid and widespread infections as one of the first European countries hit by COVID-19, forcing the government to implement strict and prolonged NPIs, such as lockdowns and curfews. These measures caused significant economic disruption, including business closures and job losses, driving up economic costs. The strict restrictions also had a profound social impact, reducing freedoms, increasing public anxiety, and contributing to high social costs [37]. Similarly, the US faced severe COVID-19 outbreaks, leading to stringent interventions and high SEC [37]. The politicization of the pandemic aggrevated these costs by reducing public compliance with health measures. Conservative politicians and media downplayed the risks of COVID-19, which led to conservatives being less compliant with protective measures [38]. This loss of public trust delayed necessary interventions, intensifying both the social and economic impact compared to countries that employed more consistent strategies.

To summarise, we calculated the SECs of the NPIs as constants based on the data from the studied period and adapted them for each country that was included in our analysis. Since SECs included in the analysis are based on certain parameters of choice (e.g. the weighted sum of social and economic cost can be altered via modifying the weights ratio), their values can also be established by researchers in subsequent studies, making this method adequate also for estimating the socioeconomic impacts of different infectious diseases.

## Results

In this section, various results and analyses are presented in relation to the uniform evaluation framework.

### Real life vs. optimal cost

**Fig 3:**
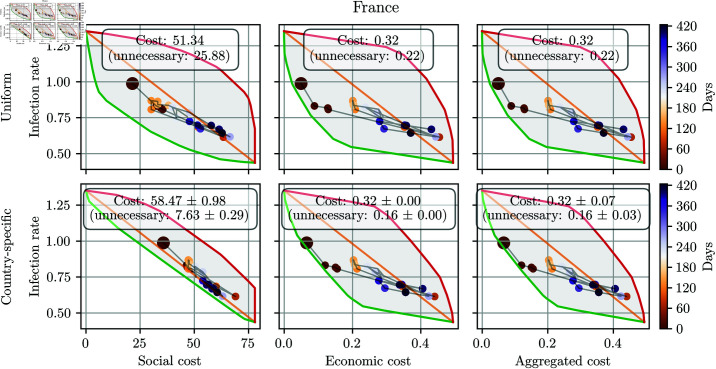
Comparison of different OxNPI configurations used in France over the studied period. The SECs were computed using uniform (first row) and country-specific (second row) stringency that was fixed over the whole studied period. In all cases, the green line depicts Pareto-optimal OxNPI configurations, while the red line represents the configurations with the worst trade-offs between the infection rate and SEC. The black dots denote NPIs implemented by decision-makers. The results are averaged over five trials. Note that the scale of the y-axis (infection rate) is the same for all the panels, while the x-axis scales vary due to the different cost units. Economic cost is shown as a relative share of GDP loss, whereas the social cost is based on domain knowledge. The aggregated cost is the average of the two costs after normalization.

**Fig 4:**
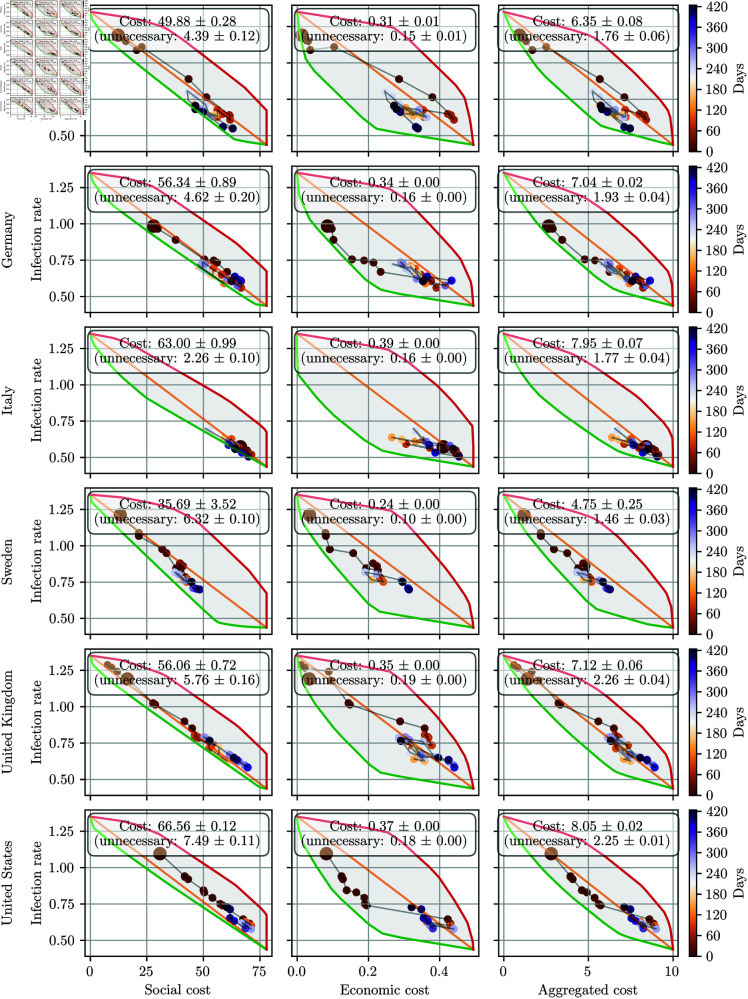
Comparison of different OxNPI configurations used by six selected countries over the studied period. Their SECs were computed using country-specific stringency that was fixed over the whole studied period. The green line depicts the Pareto front, while the red line represents the worst OxNPI configurations. The results are averaged over five trials. Economic cost is shown as a relative share of GDP loss, whereas the social cost is based on domain knowledge. The aggregated cost is the average of the two costs after normalization.

First, the costs of actually used NPIs and the computed optimal options are presented in the two-dimensional infection-cost space. Pareto fronts in Figs 3 and 4 show the trade-off between minimising infections and minimising economic costs for various countries. The Pareto front is presented as the green left curve of the object, the right red curve is presented as the inverse – worst possible option. Fig 3 illustrates Pareto-optimal OxNPI configuration sets for France, generated using the method described in the Methodology section. The upper three panels depict the fronts using uniform stringency, while the lower panels show country-specific stringency. The dots in the objects represent the NPI configurations implemented in reality, starting with mild NPIs at the beginning of the pandemic (brown dot, upper-left), followed by tightening during the first wave (dark orange dots, lower-right), relaxation (light orange dots, middle), and further tightening during the second wave (blue dots, lower-right). In every scenario, dots that are closer to the green curve represent a better outcome.

It is important to note that France and the other countries used as examples in Figs 3, 4, and later in 7 were selected purely for illustrative purposes to demonstrate patterns or trends identified by the framework. The framework is designed to apply equally to all 17 countries, and there was no intention to suggest superior or inferior performance for the countries highlighted. The selected examples are representative, and we do not imply that the performance of the framework varies significantly across different countries.

**Fig 5:**
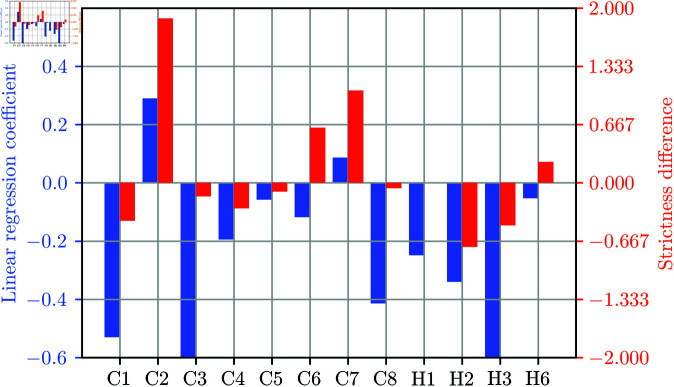
The influence of NPIs on the unnecessary costs. Blue bars (left y-axis) correspond to the NPIs’ impact to the unnecessary costs based on the linear regression coefficients, whereas the red bars (right y-axis) correspond to the mean difference between the strictness of an OxNPI implemented in real life and the strictness of the Pareto-optimal country-specific OxNPI configuration with the closest infection rate. The values were computed based on the data from all investigated countries during the study period.

**Fig 6:**
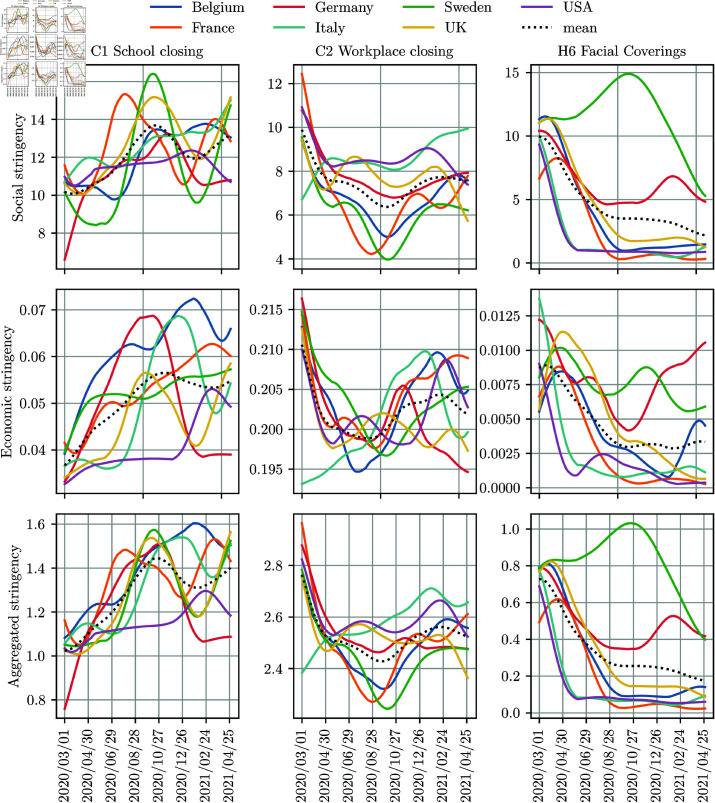
Daily country-specific time-varying stringency for seven selected countries and three OxNPIs. The first, the second, and the third column represents “C1 School closing”, “C2 Workplace closing”, and “H6 Facial coverings”, respectively. The results are averaged over five trials. Note that the y-axis limits are different for all panels.

**Fig 7:**
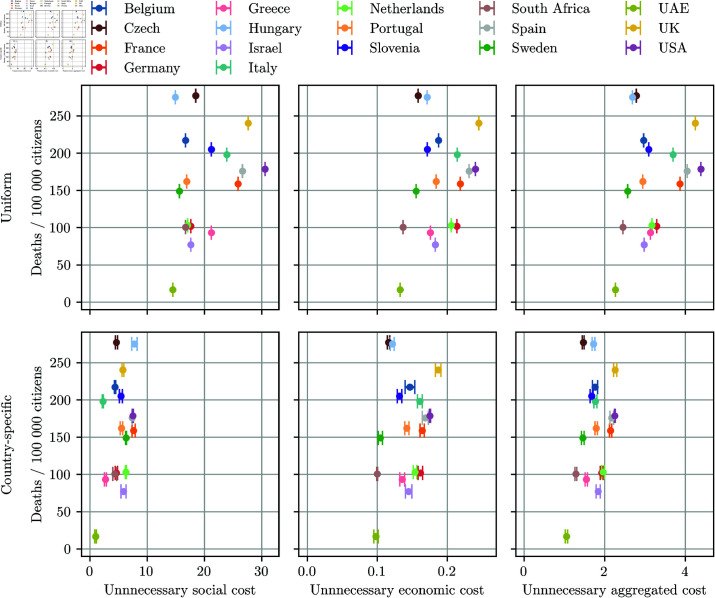
Comparison of the 17 selected countries by the number of deaths per 100k citizens vs. (top row) the mean uniform unnecessary cost and (bottom row) the mean country-specific unnecessary cost. Economic cost is shown as a relative share of GDP loss, whereas the social cost is based on domain knowledge. The results are averaged over five trials. The aggregated cost is the average of the two costs after normalization. Note that the x-axis limits are different for all panels.

As seen in Fig 3, all OxNPI configurations implemented in France in real life were suboptimal, assuming that the uniform stringency is accurate and actual infections were the same as reported. Fig 3 also highlights the unnecessary costs incurred by the country in question, indicated by the difference between any dot and the corresponding point on the green curve with the same y coordinate. This difference represents the unnecessary cost that could have been avoided by selecting an NPI combination from the Pareto-optimal set. The mean real-life and unnecessary costs are presented above each panel. From Fig 3 we can infer that considering either a uniform or country-specific stringency affects the Pareto-optimal configuration, as indicated by the green line in the two cases. By relying on country-specific calculations, however, we can also consider the country’s cultural context, which allows for a more fair evaluation of NPI cost.

Fig 4 illustrates Pareto-optimal OxNPI configuration sets for six different countries, generated using the method described in the Methodology part of this article. All panels depict the fronts calculated using country-specific stringency. As seen in the figure, all OxNPI configurations implemented in real life were suboptimal, and some were quite far from optimal.

### Unnecessary cost of implemented NPIs

As part of the evaluation framework, the unnecessary cost is evaluated as the difference between the cost of the implemented NPIs and the optimal NPIs. Table 5 shows the unnecessary costs for all 17 countries included in the analysis, taking into account country specifics (“C-s." in the table). The unnecessary cost turned out to be smaller for country-specific costs compared to uniform costs. This makes sense as uniform costs do not take into account possible cultural aspects.

**Table 5:** Mean uniform and country-specific unnecessary costs for the 17 studied countries, i.e., costs that could be avoided with different OxNPI configurations while still having the same infection rate. The country-specific stringency was fixed over the whole studied period. The county-specific costs are averaged over five trials. Italics denote the smallest and bold the largest number in a column. Economic costs are shown as a relative share of GDP loss. Abbreviations: CR, Czech Republic; UAE, United Arab Emirates; UK, United Kingdom; US, United States.

Country	Uniform	Country-spec.	Uniform	Country-spec.	Uniform	Country-spec.
	social	social	economic	economic	aggregated	aggregated
UAE	14.48	0.99 ± 0.13	0.13	0.10 ± 0.00	2.27	1.06 ± 0.00
South Africa	16.70	4.34 ± 0.35	0.14	0.10 ± 0.00	2.45	1.29 ± 0.00
CR	18.48	4.63 ± 0.18	0.16	0.12 ± 0.00	2.78	1.47 ± 0.00
Greece	21.20	2.72 ± 0.16	0.18	0.14 ± 0.00	3.14	1.55 ± 0.00
Sweden	15.62	6.32 ± 0.10	0.16	0.10 ± 0.00	2.58	1.46 ± 0.00
Slovenia	21.20	5.40 ± 0.24	0.17	0.13 ± 0.00	3.10	1.68 ± 0.00
Belgium	16.69	4.39 ± 0.12	0.19	0.15 ± 0.01	2.97	1.76 ± 0.00
Italy	23.91	2.26 ± 0.10	0.21	0.16 ± 0.00	3.70	1.77 ± 0.00
Hungary	14.92	7.76 ± 0.48	0.17	0.12 ± 0.00	2.69	1.72 ± 0.00
Portugal	16.91	5.50 ± 0.22	0.18	0.14 ± 0.00	2.95	1.79 ± 0.00
Israel	17.62	5.88 ± 0.45	0.18	0.14 ± 0.00	2.98	1.84 ± 0.00
Germany	17.61	4.62 ± 0.20	0.21	0.16 ± 0.00	3.29	1.93 ± 0.00
US	30.58	7.49 ± 0.11	0.24	0.18 ± 0.00	4.39	2.25 ± 0.00
Netherlands	17.06	6.28 ± 0.10	0.21	0.15 ± 0.00	3.18	1.96 ± 0.00
Spain	26.64	7.38 ± 0.03	0.23	0.17 ± 0.00	4.05	2.18 ± 0.00
France	25.88	7.63 ± 0.29	0.22	0.16 ± 0.00	3.87	2.15 ± 0.00
UK	27.64	5.76 ± 0.16	0.25	0.19 ± 0.00	4.25	2.26 ± 0.00

In Table 5, Italy is an interesting case because it used the costliest NPIs (probably due to one of the worst outbreaks) but paid only a modest unnecessary cost, especially when considering the country-specific SEC. Hungary, on the other hand, had the third cheapest NPIs according to the social cost but ranked highly regarding the unnecessary cost, having the highest country-specific unnecessary social cost. The United Arab Emirates used the second cheapest NPIs and paid the lowest unnecessary cost, while Sweden used the cheapest NPIs but overpaid some more. The UK used the third costliest NPIs and overpaid the most. The overall SEC is generally correlated with unnecessary costs, but there are exceptions such as Italy and Hungary. Please note that the comparisons between countries serve mainly for illustration since a range of factors and inputs influence the results; however, policymakers in each country can leverage this data for future considerations.

Next, we constructed a linear regression model with the aim of understanding which NPIs contribute the most to unnecessary costs. The strictness of individual OxNPIs was used as the predictor and the unnecessary cost as the target variable. The data for this regression model consisted of 499 OxNPI configurations implemented in real life. Blue bars in Fig 5 display linear regression coefficients, whereas red bars represent the mean difference between the strictness of an OxNPI implemented in real life and the strictness of the Pareto-optimal country-specific OxNPI configuration with the closest infection rate. A high blue bar indicates that an OxNPI contributed significantly to overpaying, while a high red bar means that it was used too frequently and/or too strictly according to the framework.

In Fig 5, “C2 Workplace closing" stands out as the largest contributor to unnecessary costs and the most overused OxNPI. This is partly because it is the most expensive one, but it also appears to have been applied less effectively. A Mann-Whitney statistical test with alpha level p = 0.05 confirmed that this OxNPI had more influence on unnecessary costs than other OxNPIs. The second-largest contributor to unnecessary costs and the second most overused OxNPI was “C7 Restrictions on internal movement." Interestingly, “C1 School closing" also contributed significantly to unnecessary costs but was underused according to the framework, suggesting inconsistent application. “H2 Testing policy" and “H3 Contact tracing" were the most underused OxNPIs, possibly due to the limited available personnel rather than the cost.

### Time varying country-specific stringency

Country-specific stringencies can be computed and observed over short time intervals, such as days. Fig 6 shows daily country-specific time-varying stringencies for three NPIs and seven selected countries. The stringencies are smoothed for presentation purposes. The first row of the figure displays the stringency for “C1 School closing." While the countries differ in their implied preferences, there is a general trend of increasing stringency for this NPI over time. The spike in cost during the summer is an anomaly, as the NPI was not in effect due to school holidays.

For “C2 Workplace closing," reluctance to implement it was high at the beginning, but most countries eventually accepted it to some degree as a necessity. Towards the end of the studied period, it was considered costlier, likely due to mounting economic damage. Some countries, such as Belgium, were more prone to closing workplaces, particularly in the middle of the study period. Italy, on the other hand, readily applied this NPI at the beginning of the study period, likely due to the severe initial impact of the pandemic. The stringency level for “H6 Facial coverings" generally decreased over time, except in the initial phase, as the measure gained widespread acceptance following some initial resistance. Notably, Sweden and Germany were among the countries most hesitant to adopt mask usage.

It is important to exercise caution when interpreting daily stringency levels, as the implementation of NPIs was influenced not only by their perceived strictness but also by the prevailing state of the pandemic. Countries tended to adopt more stringent NPIs during periods of high infection rates, and less stringent ones when cases were fewer. Another key point is that daily stringency levels fluctuate significantly and should be viewed as indicative of broader trends rather than precise daily values. The optimal time frame for accurately reflecting actual preferences in stringency levels has yet to be determined.

### Additional analysis of the 17 countries

Once the country-specific SEC and unnecessary costs are calculated, further analyses can be conducted. For instance, in this section we analyse the 17 countries that were part of the evaluation, focusing on mortality and the respective uniform and country-specific unnecessary costs. Fig 7 shows relations between COVID mortality and different types of SECs. The left column corresponds to the social, the middle to the economic, and the right column to the aggregated costs. The upper row of Fig 7 shows the uniform SEC, and the lower row the country-specific SEC incurred by the NPIs implemented in real life on the x-axis and pandemic mortality on the y-axis. The lower left corner of each square represents low mortality and low SEC, which is considered ideal but difficult to achieve, and only the United Arab Emirates managed to approach it. Countries like Greece, Germany, and Israel had higher SECs but still managed to keep the mortality rate relatively low. On the other hand, countries like Sweden had higher mortality rates but lower SECs, which can be considered a trade-off. Countries like South Africa, the Netherlands, Belgium, Slovenia, Portugal, France, Spain, and the UK had a balanced policy and were listed in decreasing order of success. The USA and Italy had high SECs without particularly low mortality rates, while Hungary and the Czech Republic had high mortality rates but still had substantial SEC.

It is important to emphasize that comparing countries is not the primary objective of either the universal evaluation framework or this paper. The main purpose is to describe an analytical tool to enhance our understanding of the effectiveness of NPIs and the timing of these interventions, thereby promoting better decision-making in future outbreaks. Furthermore, while variations in the inputs may impact the observed relationships between countries, they are unlikely to significantly alter the main conclusions from analysis of a specific country.

## Discussions and conclusions

In the event of severe infectious disease outbreaks, decision-makers are tasked with identifying the best NPIs, taking into account the disease’s characteristics and the anticipated economic and social costs. This challenge essentially involves forecasting the future, which is inherently difficult. Fortunately, there are tools available that aid in devising more effective solutions.

There exist sophisticated tools for predicting future infections and some that assess economic impacts. Nonetheless, a degree of unpredictability always remains. Assessing the outcomes after NPIs have been implemented to control an infectious disease should, in theory, not be overly complex. However, to date, there exists no comprehensive tool for this purpose. The proposed uniform evaluation framework aims to address this gap.

The framework operates under a set of assumptions and parameters. Initially, it posits that the overall financial impact can be derived from financial analyses. It allows for the input of uniform costs, requiring that these costs, when aggregated from all NPIs, reflect the overall economic impact. Country-specific costs rely on the assumption that countries rationally apply those NPIs that are best suited for their population, even though not optimally as demonstrated by several studies. The formulation of subjective SECs draws on historical decisions to reflect a specific country’s preferences. With these inputs, the algorithm identifies the Pareto front within the space of infections and SECs. A fair study of the effectiveness of the NPIs taken is thus enabled.

It is important to note that various iterations of the framework are feasible, and some have actually been explored but are not detailed here due to space constraints. In terms of infection assessment, the framework can accommodate various indicators, e.g., the number of excess deaths. Regarding costs, it can apply different economic or cost distributions, whether uniform or country-specific. This flexibility makes the framework universally applicable, regardless of the disease or country in question.

The universal evaluation framework presents multiple advantages over existing tools for this task:

a) It provides an in-depth and comprehensive analysis of the efficiency of NPIs across any infectious disease.
b) The framework accepts any amount of NPIs and costs as input.
c) It offers both uniform and country-specific evaluations of SECs.
d) It enhances decision-makers understanding of the evaluation of past NPIs, both during and after the disease’s duration.
e) The reliance on optimization algorithms and AI for evaluation and solution generation leads to a superior identification of inconsistencies in the best human-devised strategies.
f) Given its relative ease of use as a software tool, the framework could be adopted by anyone.

In conclusion, the framework appears to be innovative, providing distinct improvements in assessing the impact of previous decisions on controlling infectious diseases. It was theoretically outlined and empirically tested using COVID-19 data from the early stages of the pandemic, before widespread vaccination coverage was achieved. The software is available upon request from the authors.

One potential challenge with the proposed framework is the need for understanding AI methodologies, such as genetic algorithms or Pareto fronts. These concepts, although relatively familiar within the computing community, are often not covered in medical education. On the other hand, medical terminology and concepts might pose difficulties for AI specialists. Consequently, the formation of integrated, multidisciplinary teams is essential for a comprehensive grasp of the proposed solutions.

Future research could explore the implications of potentially disruptive interventions, such as vaccination programs. We believe that such interventions may not significantly alter the framework’s universal applicability, especially if these innovations are introduced concurrently. Nonetheless, these and other potential hypotheses require in-depth exploration and empirical validation.
